# Potential benefit of osismertinib plus bevacizumab in leptomeningeal metastasis with EGFR mutant non-small-cell lung cancer

**DOI:** 10.1186/s12967-022-03331-9

**Published:** 2022-03-14

**Authors:** Yali Yi, Jing Cai, Peng Xu, Le Xiong, Zhiqin Lu, Zhimin Zeng, Anwen Liu

**Affiliations:** 1 Department of Oncology, The Second Affiliated Hospital of Nanchang University, Nanchang, 330006 Jiangxi province China; 2 Jiangxi Key Laboratory of Clinical Translational Cancer Research, The Second Affiliated Hospital of Nanchang University, Nanchang, 330006 Jiangxi province China

**Keywords:** Leptomeningeal metastasis, EGFR-mutant, NSCLC, Osimertinib, Bevacizumab

## Abstract

**Background:**

EGFR-mutant non-small cell lung cancer (NSCLC) is prone to leptomeningeal metastasis (LM) after Tyrosine kinase inhibitors (TKIs) treatment. Our previous study suggested that osimertinib plus bevacizumab was safe and effective in LM from EGFR-mutant NSCLC. This study aimed to compare the efficacy of osimertinib plus bevacizumab with osimertinib in EGFR-mutant NSCLC patients with LM.

**Methods:**

We retrospectively reviewed the data from 27 LM patients with EGFR-mutant NSCLC who received osimertinib with or without bevacizumab at the Second Affiliated Hospital of Nanchang University. Next, we investigated the antitumor efficacy of osimertinib plus bevacizumab in an LM xenograft model using the H1975 (EGFR exon20 T790M and exon21 L858R) cell line. We examined the ability of osimertinib plus bevacizumab compared with osimertinib to penetrate the blood–brain barrier (BBB) and explored the potential mechanism.

**Results:**

Our retrospective study observed the improved survival of LM patients in osimertinib plus bevacizumab group. The median overall survival (OS) of the patients who received osimertinib and bevacizumab (n = 16) compared with osimertinib group (n = 11) was 18.0 months versus 13.7 months (log-rank test, *p* = 0.046, HR = 2.867, 95% CI 1.007–8.162). The median intracranial Progression-free Survival (iPFS) was 10.6 months versus 5.5 months (log-rank test, *p* = 0.037, HR = 3.401, 95% CI 1.079–10.720). In the LM xenograft model with H1975 cells, the combined treatment significantly increased the effective intracranial concentration of osimertinib, modulated the level of E-cadherin and downregulated the levels of EGFR and downstream signaling pathways including p-AKT and reduced tumor microvessel density (TMD), indicated that combined osimertinib with bevacizumab may exhibit a synergistic effect in EGFR-mutant LM model possibly by modulating the level of E-cadherin.

**Conclusions:**

Our findings indicate the potential benefit of osimertinib plus bevacizumab in LM with EGFR-mutant NSCLC, and more larger sample size research are still needed.

**Supplementary Information:**

The online version contains supplementary material available at 10.1186/s12967-022-03331-9.

## Introduction

Non-small cell lung cancer (NSCLC) is one of the leading causes of cancer death worldwide [1]. EGFR-mutant lung adenocarcinoma is prone to leptomeningeal metastasis (LM) after first-generation TKI treatment [2, 3]. Patients with LM have a poor prognosis and low quality of life [4, 5]. Approximately 3–5% of patients with NSCLC will develop LM, and the median OS is 4.5–11 months [6, 7]. Osimertinib, a third-generation EGFR TKI, showed efficacy superior to that of standard EGFR-TKIs in the first-line treatment of EGFR mutation advanced NSCLC and a great intracranial penetration with surprisingly high response rates [8, 9]. Osimertinib is currently the standard therapy for lung cancer metastases in the central nervous system (CNS)(including brain/leptomeningeal metastases) [10], which extended the OS to 18.8 months in LM patients with NSCLC [4, 11]. The incidence of LM in patients with lung cancer, especially in patients with EGFR mutations, is increasing with the emergence of new targeted drugs [1].

TKIs combined with antiangiogenic drugs such as bevacizumab have shown efficacy in lung cancer [12, 13]. A phase II clinical study showed that osimertinib plus bevacizumab is beneficial in the treatment of lung cancer brain metastases [14], and our previous study indicated that osimertinib plus bevacizumab was safe and effective for the treatment of LM in EGFR-mutant lung cancer [NCT04148898] [15]. LM is different from brain parenchymal metastasis, and its mechanism and treatment are complicated issues in current clinical treatment. There are currently no research data on the efficacy of osimertinib plus bevacizumab compared with osimertinib in LM.

This is the first study to compare the efficacy of osimertinib plus bevacizumab with osimertinib in LM with EGFR-mutant NSCLC through pre-clinical experiment. Aims to investigate the efficacy and potential mechanisms of osimertinib plus bevacizumab in LM with EGFR-mutant NSCLC.

## Materials and methods

### Patients

We retrospectively reviewed the charts of 27 patients diagnosed with LM from EGFR-mutant NSCLC who received osimertinib with or without bevacizumab at the Second Affiliated Hospital of Nanchang University. The date of LM diagnosis was defined as the date of first CSF cytology examination revealing malignant cells or the date of first MRI (brain or spine) demonstrating LM. The assessment of LM response was based on the modified RANO LM radiological criteria, and the CNS and extra-CNS response was evaluated according to the Response Evaluation Criteria in Solid Tumors (RECIST) version 1.1: comprehensive evaluation according to the improvement of clinical symptoms, the performance of MRI and the clearance of CSF tumor cells [16]. Given that lumbar puncture is an invasive procedure, the response criteria of LM were judged according to the improvement of clinical symptoms and the performance of MRI in our study. All LM patients underwent 1.5 T whole brain and spinal cord enhanced MRI scan at baseline, and the MRI scan thickness was 1 mm, bravo and cube sequence having a high sensitivity and specificity in the diagnosis of LM. The Overall survival (OS) was defined as the time between the initiation of diagnosis to the date of death or last follow-up by November 5, 2020. The intracranial Progression-free Survival (iPFS) was defined as the time from the diagnosis of LM to the disease progression or death. Four weeks after the initiation of osimertinib and bevacizumab, neurological evaluations, brain MRI and chest/abdominal computed tomography were routinely performed and were then performed every 1 months. The main endpoint of this study was iPFS.

The eligibility criteria were as follows: (i) age 18–80 years; (ii) histologically or cytologically confirmed NSCLC; (iii) the detection of an EGFR mutation, with EGFR status identified from primary lung tumors using the amplification refractory mutation system (ARMS) or next-generation sequencing (NGS) analysis; (iv) LM defined by CSF positivity for malignant cells and/or focal or diffuse enhancement of leptomeninges, nerve roots or the ependymal surface diagnosed by magnetic resonance imaging (MRI) with gadolinium contrast; (v) Patients receiving osimertinib plus bevacizumab or osimertinib. (vi) Patients without history of treatment with osimertinib before a diagnosis of LM. Patients who did not meet these criteria were excluded. Clinical outcomes were compared with the Kaplan–Meier log-rank test. This study was approved by the Institutional Review Board of the Second Affiliated Hospital of Nanchang University.

### Cell culture

H1975 cells (EGFR exon20 T790M and exon21 L858R) were purchased from Pro-cell in 2020 (Wuhan, China, was identified by STR) and maintained in RPMI-1640 medium supplemented with 10% Fetal Bovine Serum and 1% antibiotic solution in a humidified incubator with 5% CO_2_. All reference compounds were purchased from Gibco.

H1975 cells were stably transfected with a GV260 (GeneChem, Shanghai, China) vector containing luciferase, and bioluminescence signals were measured by an in vivo imaging system. H1975-luc tumor cells were prepared for injection after trypsinization and washing with PBS. A viable cell count was performed with trypan blue to adjust the cell concentration to 2 × 10^6^ cells in PBS for each injection.

### Animal model of LM

BALB/C nude female mice (6–8 weeks) were obtained from institutional animal breeding services (the Nanchang Royo Biotech Co., Ltd.). The animals were group housed five a cage in a temperature-controlled room on a 12-h/12-h light/dark cycle with unlimited access to food and water. All animal procedures in this study were approved by the Animal Care and Use Committee of Nanchang University, and ethical approval was obtained from the Institutional Review Board of the Second Affiliated Hospital of Nanchang University.

Mice were anesthetized with 4% isoflurane. The skull was exposed with a skin incision (1 cm) under sterile conditions (ethanol skin wipe) to locate the bregma. A Hamilton syringe needle with H1975-luc human NSCLC cells was injected into the right lateral ventricle (anteroposterior 2.0 mm from the bregma; lateral 0.2 mm to the right; and dorsoventral 4 mm) at 2 μl per min. A total volume of 5 μl of cell suspension was injected [17, 18]. The tumor burden of intracranial lesions and the tumor size in the pia mater were measured using a BLI technique with an in vivo imaging system, MRI and H&E staining.

### In vivo pharmacodynamic study

After confirming tumor formation, the mice were randomly divided into 4 groups (n = 9 per group): the control(0.9% normal saline, daily, oral), osimertinib (25 mg/kg, daily, oral) [19], and bevacizumab (5 mg/kg twice a week, i.p.) [20], and osimertinib plus bevacizumab. Brain tissues were collected at 1, 6, 12, and 24 h after a single dose of treatment to determine the penetration of osimertinib in the brain with a validated liquid chromatography-tandem mass spectrometry method (n = 3). Furthermore, tumor tissues were collected in formalin or frozen at the endpoint of experiments, EGFR downstream signals were evaluated by immunoblotting, and an angiogenesis marker (CD31) was evaluated by immunofluorescence. Once the nude mice lost more than 20% of their weight, the experiment reached the endpoint, and the mice were euthanized by intraperitoneal injection of pentobarbital sodium (150 mg/kg). Each experimental group has at least 9 mice, and a total of 40 mice are included.

### Formaldehyde perfusion of the brain

To keep the mouse brain tissue as intact as possible, this experiment used formaldehyde perfusion to fix the brain. The mice were anesthetized by the intraperitoneal injection of 1% pentobarbital sodium (50 mg/kg), the chest skin was cut open with scissors, the heart was exposed, a syringe needle was inserted into the left ventricle from the left apex towards the aorta, the needle was fixed with hemostatic forceps, physiological saline was quickly perfused until the liver tissue color turned gray, and then paraformaldehyde was slowly perfused. The mouse limbs twitched, and the whole body became stiff. After successful perfusion, the mouse brain tissue was removed and fixed with paraformaldehyde for subsequent experiments.

### Histology

#### H&E staining and immunofluorescence

Mouse brain tissues were quickly excised, immersed in 10% paraformaldehyde, and embedded in paraffin. Brain tissues were cut into 5-μm thick sections. Slides were stained with hematoxylin and eosin (H&E). Pictures were obtained with × 10 and × 40 objective respectively, the pathology diagnosis was completed by two independent pathology doctors. Tumor microvessel density (TMD) was measured by staining for CD31 (Proteintech, Wuhan, China), as previously reported [21]. Briefly, TMD was assessed in hot spots of brain tissue cross-sections identified by light microscopy. Five equal areas were then photographed with a 40 × objective (400× magnification). The staining was scored by two independent and experienced pathologists and calculated as the product of the staining intensity. The areas and integrated optical density (IOD) of the slides were analyzed by Image-Pro Plus 6.0 software.

#### Western blotting

Western blotting was performed according to standard methods, as described previously [21]. The tumor sections in brain tissue were snap-frozen in liquid nitrogen for protein isolation, and then tumors were homogenized with a mortar and pestle and lysed in RIPA buffer containing Halt protease and phosphatase inhibitor cocktail. Soluble proteins were quantified by a BCA protein level detection kit and then subjected to SDS-PAGE followed by immunoblotting. Antibody incubation was conducted overnight at 4 °C. Antibodies included EGFR (1:1000, Servicebio, GB11084-2), AKT (1:1000, Servicebio, GB11689), S473 p-AKT (1:1000, Affinity, AF0832), ERK (1/2) (1:1000, Servicebio, GB11560), p-ERK (1/2) (1:1000, Cell Signaling Technology, #4370), E-cadherin (1:1000, Servicebio, GB14076), HIF-1α (1:1000, Servicebio, GB111339), ACTIN (1:3000, Servicebio, GB12001). Secondary antibodies were diluted 1:5000, and immunoreactive proteins were visualized using a Western blotting machine (Thermo) and analyzed with ImageJ software.

#### IHC

Tumor-bearing brain tissue were collected from mice of each group. Tissues were fixed in 10% buffered formalin for 24 h following standard procedure for processing, paraffin-embedding, and sectioning to assess PCNA (1:200, Servicebio, GB11010), E-cadherin (1:200, Servicebio, GB14076) by IHC assay. The reaction was visualized using the Servicebio image analysis system, the staining was scored by two independent and experienced pathologists and calculated as the product of the staining intensity. First divides the positive grade: negative without staining, score 0, weak positive light yellow, score 1, medium positive brown, score 2, strong positive brown, score 3 points. Then analyze and calculate the area of weak, medium, and strong positive in the measurement area, the tissue area of the measurement area, the cumulative optical density value of the positive and the positive area. PCNA and E-cadherin quantification for each sample was determined and molecular data using modified H-scores ([{% of weak staining} × 1] + [{% of moderate staining} × 2] + [{% of strong staining} × 3]), to determine the overall percentage of PCNA and E-cad positivity across the entire stained sample, yielding a range from 0 to 300 [22, 23].

### Statistics

Preclinical data are presented as the mean ± SD. Data were analyzed by one-way ANOVA, followed by Dunnett’s test or Student’s t-test. Survival analysis of animal was performed using a Kaplan–Meier survival curve and a log-rank test. The Kaplan–Meier method was used to estimate and graphically present OS and iPFS. All analyses were performed using GraphPad Prism 7.0 (GraphPad Prism Software, Inc) or IBM SPSS statistics 22. Experiments were repeated independently at least three times. *p* < 0.05 was considered statistically significant.

## Results

### Osimertinib plus bevacizumab may improve the efficacy of LM patients with EGFR-mutant NSCLC

**Fig. 1:**
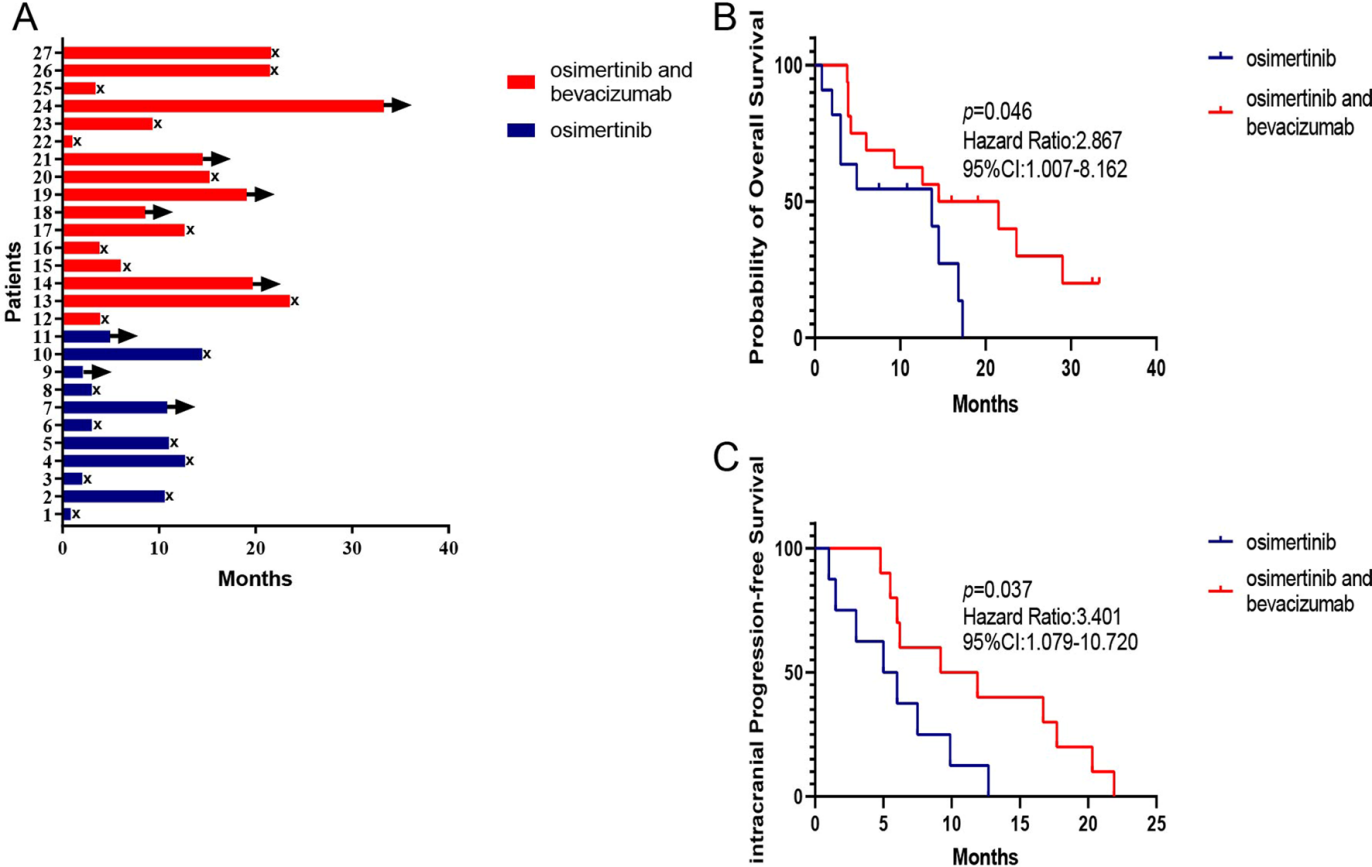
Swimmer plot and OS and iPFS curve of LM patients with EGFR mutant NSCLC**.** **A** Swimmer plot of LM patients with EGFR mutant NSCLC received osimertinib with or without bevacizumab. → means alive, x means dead. **B** OS of osimertinib plus bevacizumab group and osimertinib group (log-rank test, *p* = 0.046). **C** iPFS of osimertinib plus bevacizumab group and osimertinib group (log-rank test, *p* = 0.037)

A total of 70 patients diagnosed with lung cancer and LM in at the Second Affiliated Hospital of Nanchang University from October 2017 to March 2020 were collected. 27 patients with EGFR mutation-positive NSCLC with LM were selected according to the inclusion criteria (Table 1). Enrolled patients received osimertinib 80 mg orally daily with or without bevacizumab 7.5 mg/kg intravenously every three weeks. Treatment continued until the disease progressed, unacceptable adverse events occurred, or the patient withdrew consent. The pathological type of all patients is lung adenocarcinoma. In these patients, EGFR mutations included L858R (51.8%) (1 T790M), exon 19 deletion (48%) (2 T790M). The median follow-up time was 19.1 months. In osimertinib group, Of the 11 total patients, 81.8% (9) had clinical response, 3 of patients achieved partial response (PR) (27.3%), 6 of patients had stable disease (SD), 2 of patients had progressive disease (PD). In osimertinib plus bevacizumab group, Of the 16 total patients, 81.3% (13) had a clinical response, 6 of patients achieved PR (37.5%),7 of patients had SD, 3 of patients had PD. The assessments of response to osimertinib or osimertinib plus bevacizumab in 27 LM patients were shown in Additional file 1: Table S1 and Table S2. The other metastasis sites of patients were shown in Additional file 1: Table S3. The Swimmer plot was accordance with treatment duration of 27 LM patients (Fig. 1A). There is an increasing of median OS of the patients who received osimertinib and bevacizumab (n = 16, 18.0 months) as compared with patients with osimertinib treatment (n = 11, 13.7 months) (log-rank test, *p* = 0.046, HR = 2.867, 95%CI 1.007–8.162). The median iPFS is 10.6 months versus 5.5 months (log-rank test, *p* = 0.037, HR = 3.401, 95%CI 1.079–10.720) (Fig. 1B and C). These data suggested that plus bevacizumab may improve the survival time of patients with LM from EGFR mutant NSCLC.

**Table 1:** Patient characteristics (n = 27)

Characteristics	Osimertinib (n = 11) %	Osimertinib and bevacizumab (n = 16) %
Age (median)	47–70 (56)	46–75 (60)
Gender		
Female	4 (36.4)	8 (50.0)
Male	7 (63.6)	8 (50.0)
History of tobacco exposure		
Yes	6 (54.5)	7 (43.8)
No	5 (45.5)	9 (56.2)
ECOG performance status score		
≥ 2	9 (81.8)	11 (68.8)
< 2	2 (18.2)	5 (31.2)
EGFR mutation		
Exon 21 L858R	6 (54.5) (1 T790M)	8 (50.0)
Exon 19 deletion	5 (45.5) (1 T790M)	8 (50.0) (1 T790M)
Neurological symptoms		
Yes	10 (90.9)	15 (93.8)
No	1 (9.1)	1 (6.2)
Exclusively diagnosed LM by CSF cytology		
Yes	5 (45.5)	8 (50.0)
No	6 (54.5)	8 (50.0)
Exclusively diagnosed LM by MRI		
Positive	9 (81.8)	12 (75.0)
Negative	2 (18.2)	4 (25.0)
Both positive for MRI and CSF cytology		
Yes	5 (45.5)	5 (31.2)
No	6 (54.5)	11 (68.8)
Systemic therapy before LM diagnosis		
First generation TKIs		
Yes	10 (90.9)	14 (87.5)
No	1 (9.1)	2 (12.5)
Chemotherapy		
Yes	1 (9.1)	3 (18.7)
No	10 (90.9)	13 (81.3)
Brain radiotherapy		
Yes	2 (18.2)	2 (12.5)
No	9 (81.8)	14 (87.5)
Subsequent treatments after osi ± beva		
Osimertinib + ITC	1 (9.1)	3 (18.8)
Osimertinib + WBRT	0 (0.0)	1 (6.2)
Chemotherapy	2 (18.2)	1 (6.2)
Osimertinib 160 mg	1 (9.1)	0 (0.0)
Continued prior treatments	7 (63.6)	11 (68.8)

*CSF* cerebrospinal fluid, *ECOG* Eastern Cooperative Oncology Group, *EGFR* epidermal growth factor receptor; *LM* leptomeningeal metastasis, *MRI* magnetic resonance imaging, *TKIs* tyrosine kinase inhibitors, *osi ± beva* osimertinib with or without bevacizumab, *ITC* intrathecal chemotherapy, *WBRT* whole-brain radiotherapy

### Osimertinib plus bevacizumab demonstrated impressive CNS penetration in vivo

**Fig. 2:**
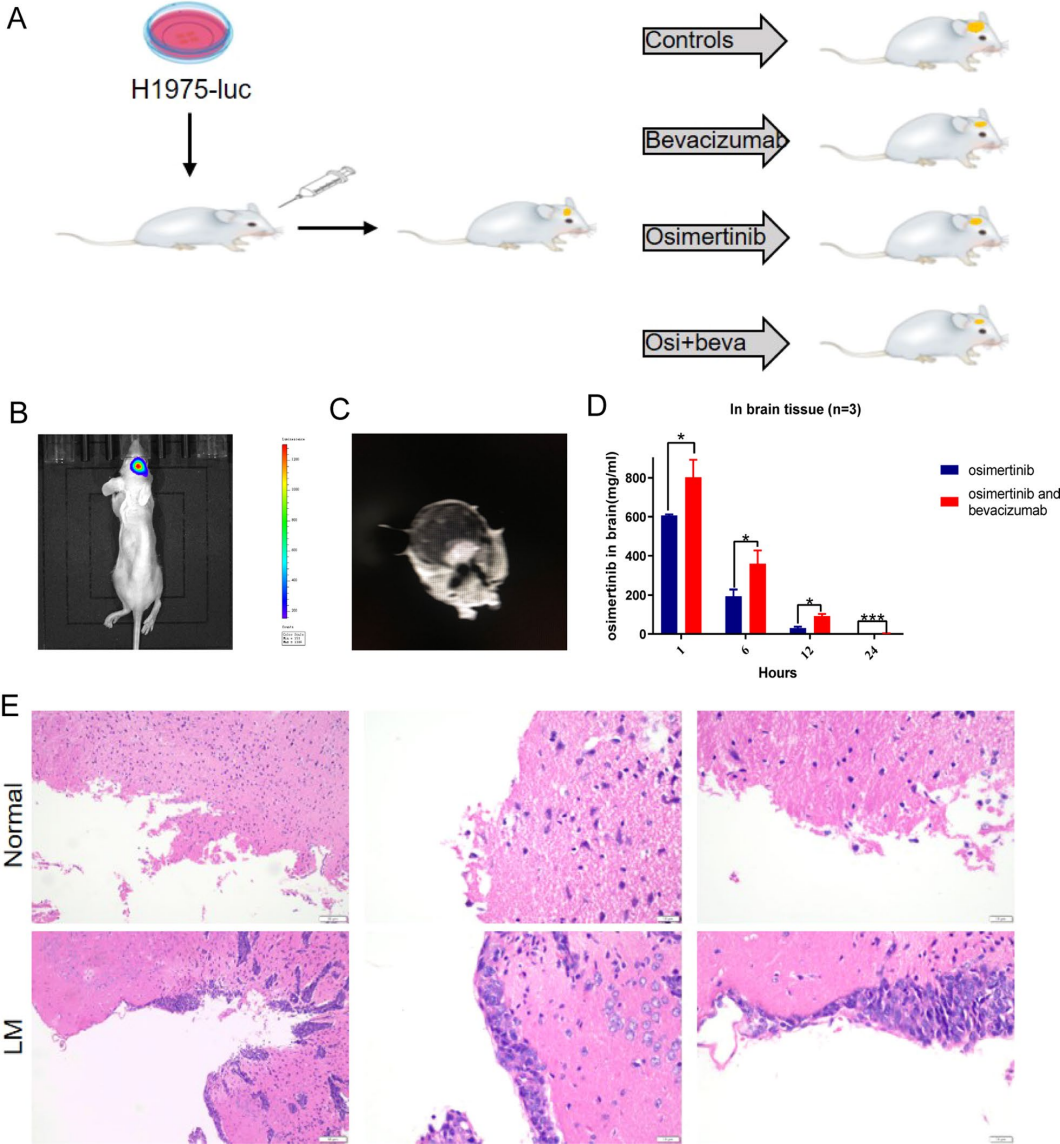
Construction of LM model and short-term oral absorption test in vivo. **A** Flow chart of LM model. H1975-luc cells were injected through the right ventricle. Representative images of **B** IVIS imaging, **C** MRI at week 2 after injection tumor cells, and **E** the leptomeningeal tissue of normal group and LM group was compared by H&E staining. **D** Concentration of osimertinib in the mouse brain tissue in 1,6,12,24 h after single dose of osimertinib with or without bevacizumab (n = 3, * means *p* < 0.05, *** means *p* < 0.001)

To determine the efficacy of osimertinib plus bevacizumab in LM in EGFR-mutant NSCLC, we constructed a model of LM with lateral ventricle injection (Fig. 2A), and the lung cancer LM xenograft model was confirmed by IVIS imaging, MRI, and H&E (Fig. 2B, C and E). Mass spectrometry analysis showed that the average concentration of osimertinib in the brain tissue in combined group was higher than osimertinib group [ 803.2 vs 606.5 ng/ml (1 h after treatments), 359.6 vs 193.8 ng/ml (6 h), 91.8 vs 31.2 ng/ml (12 h), 3.8 vs 1.0 ng/ml (24 h) (n = 3 per group, *p* < 0.05)] (Fig. 2D). These results suggested that when osimertinib is combined with bevacizumab, the concentration of osimertinib in the mouse brain was increased.

### Osimertinib plus bevacizumab effectively inhibits the growth of EGFR-mutant LM xenografts in nude mice

**Fig. 3:**
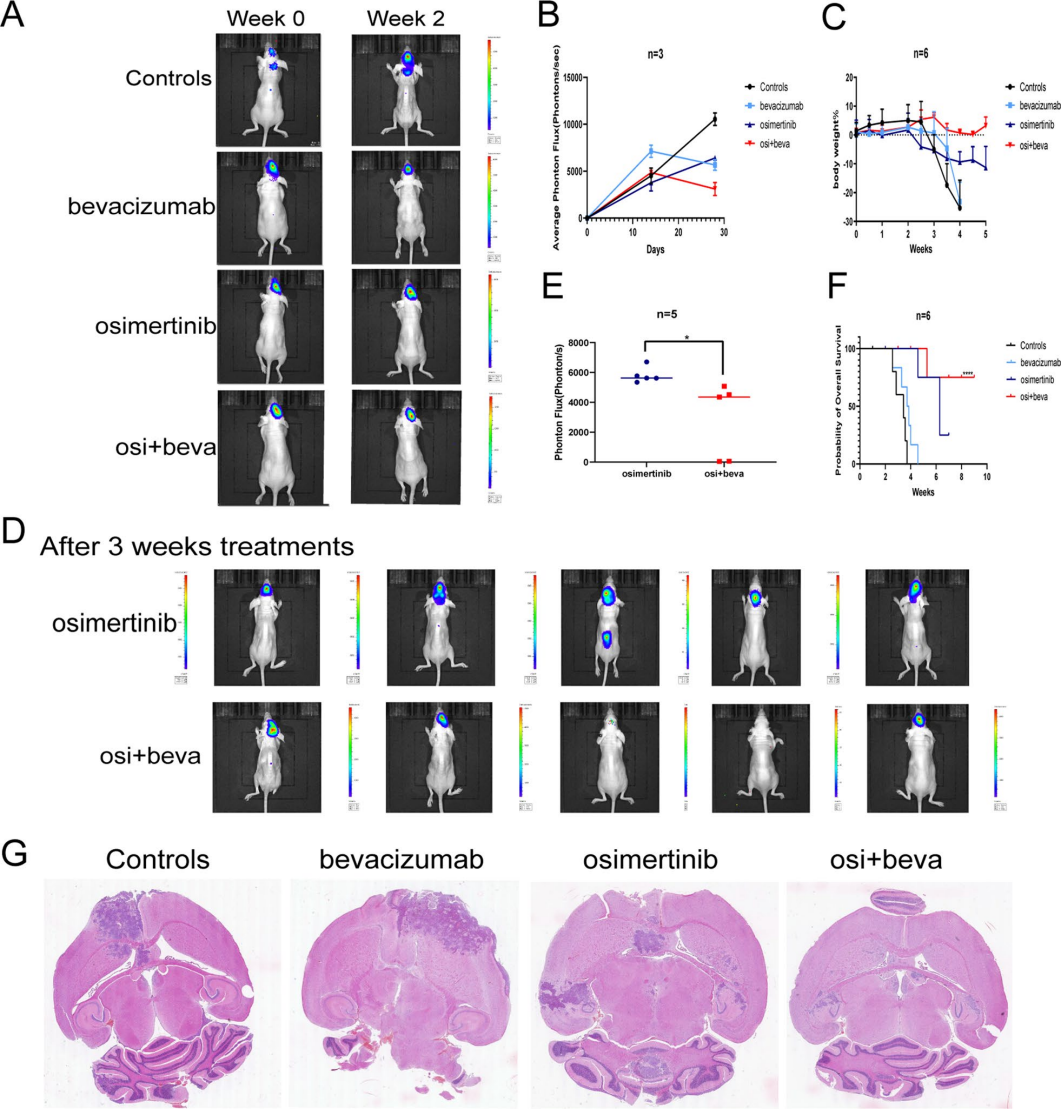
Antitumor activity of osimertinib and bevacizumab in the EGFR-mutant LM model **A**. Representative images of luciferin signals at weeks 0 and 2, significantly lower luciferin signals were detected in osimertinib and bevacizumab-treated animals compared with other groups after 2 weeks treatments. **B** The quantification of luciferin signals of (**A**) (*p* < 0.05). **C** Body weight changes in four groups of LM xenografts model (n = 6). **D** IVIS imaging of the osimertinib group and osimertinib plus bevacizumab group after 3 weeks treatments. **E** The quantification of luciferin signals of (**D**). **F** Survival curves of four groups (n = 6, log-rank test, **** means *p* < 0.0001). **G** The H&E staining for the four groups

The effects of osimertinib plus bevacizumab in the LM xenograft model were validated. Within 2 weeks of treatment, the three groups had different degrees of tumor regression except for the progression of the control group compared to baseline (Fig. 3A and B). The body weight change and survival curves implied that the tumor regression of the combined group was superior to that of the osimertinib group after 3 weeks of treatment. The mice in the bevacizumab group lost a significant amount of weight and died quickly, like mice in the control group. (n = 6 per group, *p* < 0.05) (Fig. 3C and F). Compared with that in the osimertinib group, the tumors in the osimertinib plus bevacizumab group were significantly regressed (Fig. 3D and G). These results suggested that the combination effectively inhibits the growth of EGFR-mutant LM tumors in *vivo*.

### Osimertinib plus bevacizumab suppresses the EGFR downstream signaling pathway and modulates E-cadherin levels in EGFR-mutant LM model mice

**Fig. 4:**
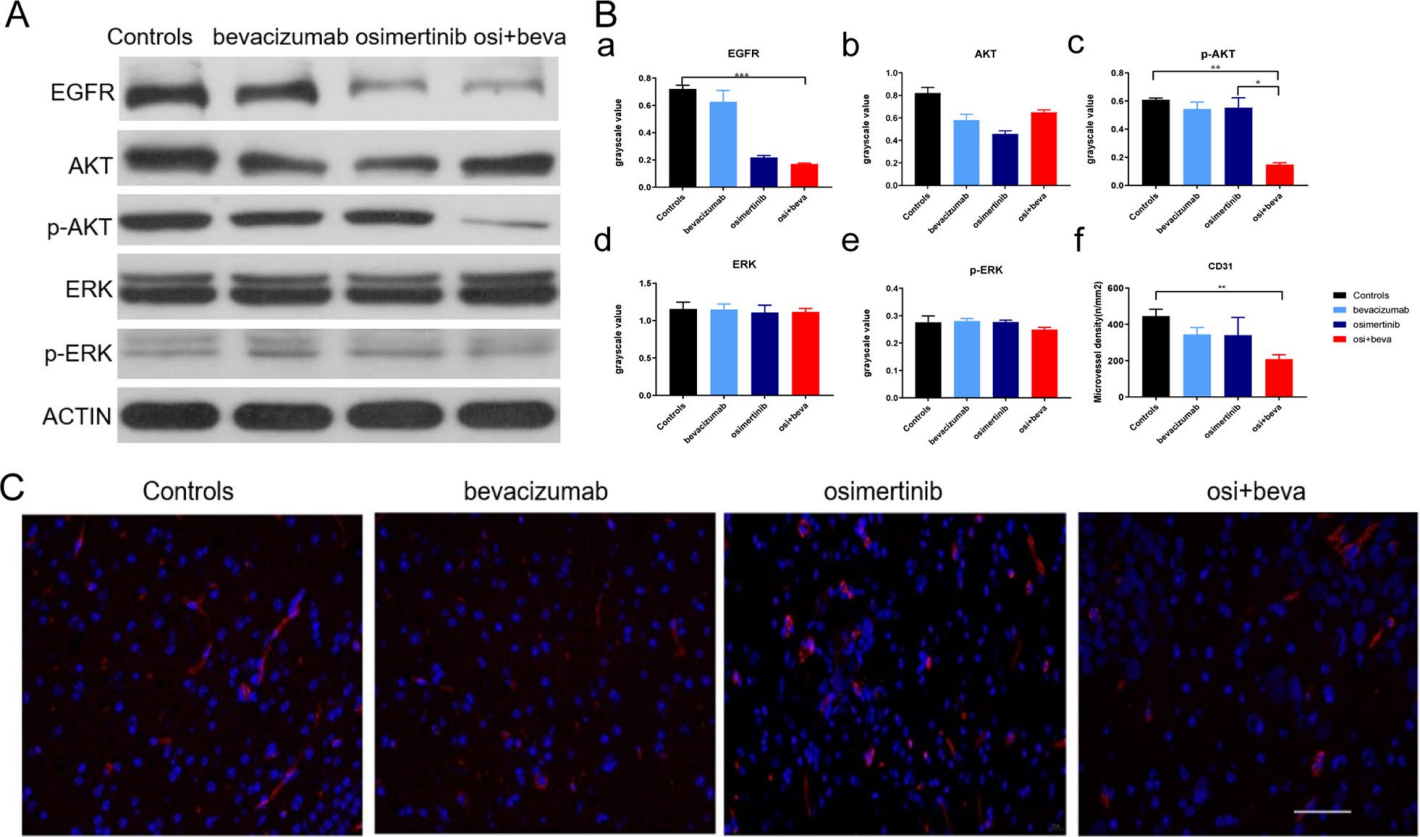
Osimertinib plus bevacizumab suppresses the EGFR downstream signaling pathway and reduce the TMD** A** AKT, p-AKT, ERK, p-ERK, EGFR were defected in the tumor tissues of four groups by WB. **B**. a–e grayscale value of AKT, p-AKT, ERK, p-ERK, EGFR. f Quantification of CD31 of fluorescence (n = 3, *p* < 0.05, one-way ANOVA). **C** Tumor blood vessel density fluorescence results and histogram(red color, CD31; Green color, nuclear)(n = 3,**means *p* < 0.01, one-way ANOVA). Scale bars, 400 μm. Osi + beva means osimertinib plus bevacizumab

**Fig. 5:**
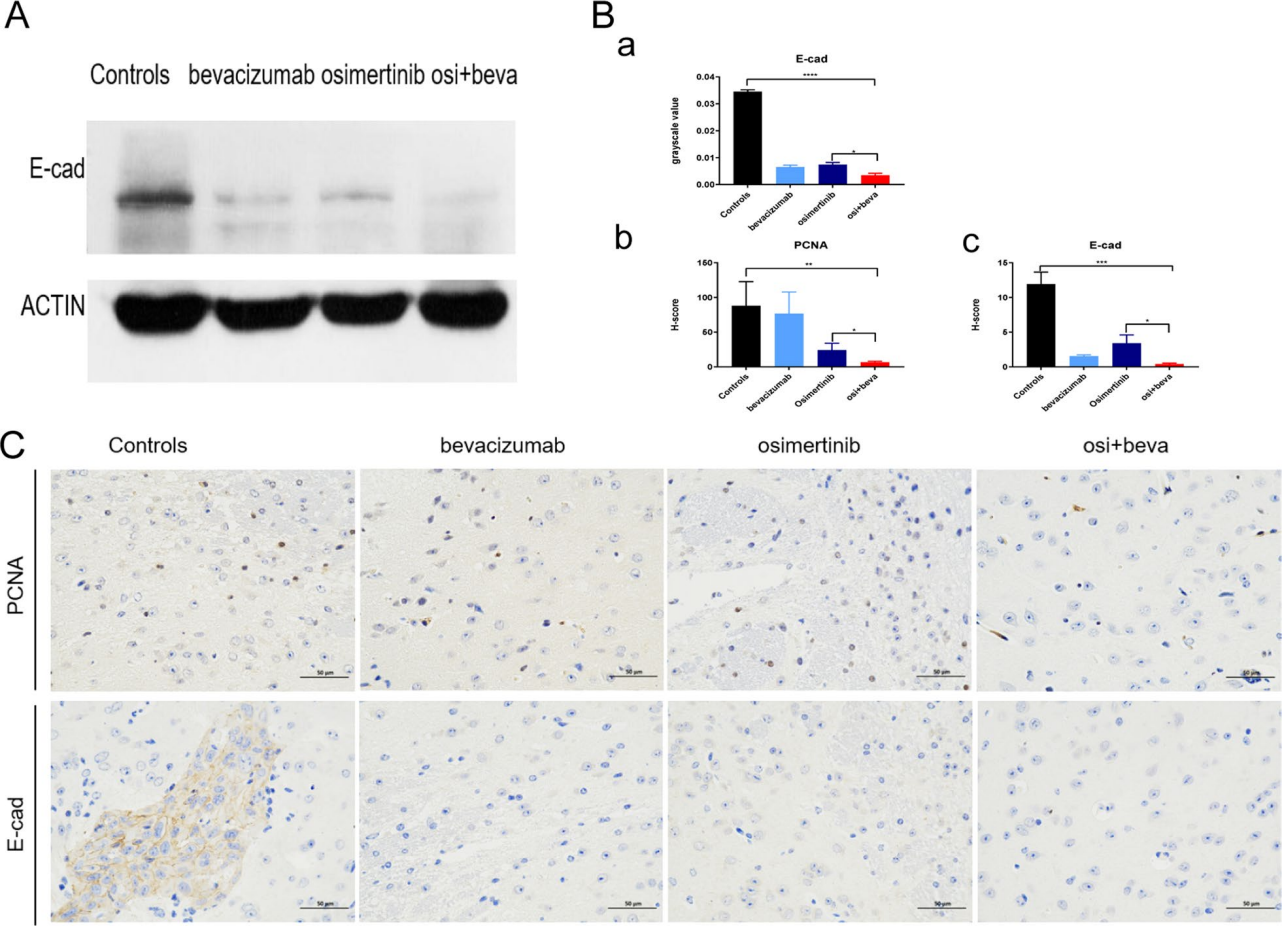
Osimertinib plus bevacizumab modulates E-cadherin levels in EGFR-mutant LM model mice. **A** E-cadherin were assessed in the tumor tissues by WB. **B** grayscale value of E-cadherin (a), quantification of PCNA (b) and E-cadherin (c) by IHC (d). **C** PCNA, E-cadherin were assessed in the tumor tissues by IHC. (n = 3**,** *means *p* < 0.05, **means *p* < 0.01, *** means *p* < 0.001, **** means *p* < 0.0001, one-way ANOVA**)**

The EGFR downstream signaling pathway was examined in tumor tissues by Western blotting. The results showed that p-AKT and EGFR were significantly decreased in the osimertinib plus bevacizumab group compared with the other groups. (n = 3, *p* < 0.05) (Fig. 4A and B a–e). Meanwhile, our data showed that TMD was significantly decreased in the osimertinib and bevacizumab group compared with that in the other groups (n = 3, *p* < 0.05) (Fig. 4C and B f). These results further verified that osimertinib and bevacizumab could play a synergistic effect in EGFR-mutant LM model. To understand the potentially mechanism of the combination treatment, we assessed PCNA, E-cadherin, ADAM9 and HIF-1α levels in xenograft tumors receiving the treatments. The combination did not affect the levels of ADAM9 and HIF-1α, but the E-cad levels was more significantly decreased in combination group compared with osimertinib group (n = 3, *p* < 0.05) (Fig. 5). Hence, we demonstrated the in vivo modulation of E-cadherin by the combination of osimertinib and bevacizumab.

## Discussion

Due to the unclear mechanism of LM and the existence of the blood–brain barrier, it is difficult for drugs to reach an effective intracranial concentration. The treatment of LM in lung cancer is still a complicated problem. This is the first pre-clinical study to compare the effects of osimertinib with or without bevacizumab in LM of EGFR-mutant NSCLC. The efficacy is compared through a combination of basic research and clinical analysis and its possible mechanism is explored.

Studies have shown that EGFR-TKIs (erlotinib) and angiogenesis inhibitors that target endothelial growth factor receptor (anti-VEGFR) (bevacizumab) (A + T) achieved superior PFS and acceptable safety in NSCLC patients with intracranial metastasis [24]. Our study retrospectively analyzed 27 LM patients with EGFR-mutant lung cancer who received osimertinib with or without bevacizumab, the median OS of osimertinib plus bevacizumab group (n = 16) compared osimertinib group (n = 11) was 18.0 months versus 13.7 months (log-rank test, *p* = 0.046, HR = 2.867, 95%CI 1.007–8.162). The median iPFS is 10.6 months versus 5.5 months (log-rank test, *p* = 0.037, HR = 3.401, 95%CI 1.079–10.720). Then we investigated the antitumor effects of osimertinib and bevacizumab in an EGFR-mutant LM model. We found that osimertinib plus bevacizumab significantly improved the concentration of osimertinib in mouse brain tissue.

A series of studies have supported that A + T therapy improved survival benefits [25]. The JO25567 and NEJ026 [13] reported that the OS and PFS in A + T therapy were prolonged and recommended combined erlotinib and bevacizumab as a first-line regimen in EGFR mutation-positive NSCLC [26, 27]. The PFS of NSCLC patients with pleural or pericardial effusion is expected to be prolonged with osimertinib plus bevacizumab and to demonstrate their safety [28]. Consistent with the above studies, our preclinical experiments and retrospective analysis indicated that osimertinib and bevacizumab may improve the survival of LM patients with EGFR mutant NSCLC. In contrast, some reports showed that TKIs combined with angiogenesis inhibitors did not improve PFS in EGFR-mutant NSCLC [29]. WJOG 8517L reported that although the overall response rate (ORR) was better with osimertinib plus bevacizumab than osimertinib alone (68% vs 54%), median PFS was not longer with osimertinib plus bevacizumab (9.4 months vs 13.5 months) and median OS have no difference (not reached vs 11.2 months) [30]. In addition, although no difference in PFS was observed between osimertinib plus bevacizumab and osimertinib alone. subgroup analyses from ETOP-booster suggested that addition of VEGF inhibitor is beneficial in smokers only [31]. However, there was no statistical difference in OS and iPFS among the smokers in our study, the sample size was too small to be counted. It is an interesting study on the potential relationship between TP53 and bevacizumab which deserves further research in the future. Generally, LM or CNS metastasis was excluded from the clinical studies. The clinical benefits of combined therapy in patients with LM still not clear.

VEGF is a key regulator of angiogenesis and a validated target for NSCLC [32]. The biologically synergistic antitumor activity of EGFR inhibition in combination with VEGF/VEGFR pathway blockade have been demonstrated in preclinical studies [33]. In EGFR-mutant NSCLCs, up-regulated EGFR signaling increases VEGF through hypoxia-independent mechanisms, and elevated VEGF, in turn, contributes to the emergence of resistance to EGFR tyrosine kinase inhibitors (TKIs) [34], and EGFR, similar to VEGFR-2, can be expressed on tumor-associated endothelial cells [33]. The inhibitory effects of afatinib on EMT and tumorigenesis may be associated with the ERK‑VEGF/MMP9 signaling pathway [35]. The TMD has been regarded as one important indicator for quantitatively analyzing tumor angiogenesis, which can clearly reflect the intra-tumoral blood vessels state and tumor-induced angiogenesis ability [36, 37]. Tumor vascularization is critical to the pathogenesis of solid tumors, and TMD is related to tumor invasiveness and metastasis formation which could be used as a potential predictive marker for bevacizumab benefit [38]. This antagonistic effect of VEGF inhibit immature angiogenesis and induce vascular normalization, thus increasing the internal perfusion of the tumor and increasing the rate of drug delivery [39]. In theory, bevacizumab increases the rate of drug entry into the brain, combined with osimertinib possibly have more advantageous in the control of intracranial lesions. Considering, our study found that the combined treatment significantly increased the effective intracranial concentration of osimertinib, modulated the level of E-cadherin and downregulated the levels of EGFR and downstream signaling pathways including p-AKT and reduced TMD, indicated that combined osimertinib with bevacizumab could play a synergistic effect in EGFR-mutant LM model possibly by modulating the level of E-cadherin.

Although the current study demonstrated that osimertinib plus bevacizumab was benefit for LM with EGFR mutation NSCLC. Given the limitation of our analysis, these results are hypothesis generating and should be interpreted with caution. First, strictly speaking, our LM model belongs to a local growth model not metastasis model. Currently, there is no stable disease model that metastasizes to the meninges via the primary tumor. Commonly used models including cisternal injection and lateral ventricle injection, both of which are spread to the meninges through the cerebrospinal fluid circulation route. They have not been metastasized from the primary focus. Hence, they are not suitable for the study of the mechanism of LM. However, due to their excellent performance stability, it is benefit for drug intervention experiments. Second, it is a small sample size retrospective study. It is difficult to assess the iPFS of LM, and it is currently believed that a comprehensive assessment should be based on patient neurological examination, C radiological evaluation, and cerebrospinal fluid cytology. The main challenge is to define measurable and non-measurable (target) damage, and allow assessment of response changes, perform cerebrospinal fluid cytology for assessment, due to lumbar puncture is an invasive test, most patients refuse to perform. Therefore, it is difficult and subjective to evaluate iPFS in patients with LM, and the present results must be interpreted cautiously. The data were obtained from medical files, and we cannot exclude the possibility of undefined biases and/or confounding factors. Third, the interaction mechanism of osimertinib and bevacizumab needed to be further explored. The next step of our research, the phase II study of osimertinib plus bevacizumab for LM is already ongoing (NCT04425681). We are collecting the CSF and blood of NSCLC patients with LM in osimertinib group and the combination with bevacizumab group to further explore the mechanism and find the biomarkers for prognostic.

In conclusion, the current findings demonstrated the potential benefit of osimertinib plus bevacizumab in LM with EGFR mutant NSCLC, and more larger sample size research are still needed.

## Supplementary Information

**Additional file 1**: **Table S1.** The assessment of response for 27 LM patients. **Table S2.** The response for 27 patients. **Table S3.** The extracranial metastasis of 27 patients

## Data Availability

All data generated or analyzed during this study are included in this published article.

## Abbreviations

EGFR: Epidermal growth factor receptor
H&E: Hematoxylin–eosin staining
iPFS: Intracranial progressive-free survival
IVIS: Interactive video information system
LM: Leptomeningeal metastasis
MRI: Magnetic resonance imaging
NSCLC: Non-small cell lung cancer
TKIs: Tyrosine kinase inhibitors
TMD: Tumor microvessel density
OS: Overall survival
