# Correction: Potential benefit of osimertinib plus bevacizumab in leptomeningeal metastasis with EGFR mutant non-small-cell lung cancer

**DOI:** 10.1186/s12967-022-03453-0

**Published:** 2022-06-27

**Authors:** Yali Yi, Jing Cai, Peng Xu, Le Xiong, Zhiqin Lu, Zhimin Zeng, Anwen Liu

**Affiliations:** 1 Department of Oncology, The Second Affiliated Hospital of Nanchang University, Nanchang, 330006 Jiangxi Province China; 2 Jiangxi Key Laboratory of Clinical Translational Cancer Research, The Second Affiliated Hospital of Nanchang University, Nanchang, 330006 Jiangxi Province China

## Correction to: Journal of Translational Medicine (2022) 20:122 10.1186/s12967-022-03331-9

Following publication of the original article [1], we have been notified that there is an error in the title of the article. The correct title should as per below:

Potential benefit of osimertinib plus bevacizumab in leptomeningeal metastasis with EGFR mutant non-small-cell lung cancer.

The original article has been corrected.
