# Supplementary material for: Potential benefit of osismertinib plus bevacizumab in leptomeningeal metastasis with EGFR mutant non-small-cell lung cancer
Source: J Transl Med. 2022 Mar 14;20:122. doi: 10.1186/s12967-022-03331-9 (PMC8919569; doi:10.1186/s12967-022-03331-9)
Supplement: Supplementary file 1 — Additional file 1: Table S1. The assessment of response for 27 LM patients. Table S2. The response for 27 patients. Table S3. The extracranial metastasis of 27 patients [file 12967_2022_3331_MOESM1_ESM.docx]

**Table S1. the assessment of response for 27 LM patients.**

| Patient | CNS symptoms | MRI Image | CSF cytology | Response | Subsequent treatment |
| --- | --- | --- | --- | --- | --- |
| 1 | Worse | Not done | Not done | Progression | Best supportive care |
| 2 | Worse | Stable | Stable | Stable | Osimertinib 160mg |
| 3 | Worse | Not done | Stable | Progression | Best supportive care |
| 4 | Stable | Stable | Not done | Stable | chemotherapy |
| 5 | Improved | Improved | Not done | Response | chemotherapy |
| 6 | Stable | Stable | Not done | Stable | - |
| 7 | Stable | Improved | Not done | Response | - |
| 8 | Stable | Not done | Not done | Stable | - |
| 9 | Stable | Stable | Not done | Stable | Osi+ITC |
| 10 | Stable | Improved | Not done | Response | - |
| 11 | Stable | Not done | Not done | Stable | - |
| 12 | Stable | Worse | Not done | Progression | - |
| 13 | Worse | Improved | Not done | Stable | - |
| 14 | Improved | Stable | Stable | Stable | Osi+ITC |
| 15 | Stable | Stable | Not done | Stable | - |
| 16 | Stable | Not done | Not done | Stable | - |
| 17 | Stable | Negative | Not done | Response | - |
| 18 | Improved | Worse | Not done | Response | Osi+ WBRT |
| 19 | Improved | Improved | Not done | Response | - |
| 20 | Stable | Improved | Not done | Response | - |
| 21 | Stable | Negative | Not done | Response | - |
| 22 | Stable | Improved | Not done | Response | - |
| 23 | Worse | Not done | Not done | Progression | Osi+ITC |
| 24 | Stable | Not done | Not done | Stable | - |
| 25 | Stable | Stable | Negative | Stable | Osi+ITC |
| 26 | Stable | Stable | Not done | Stable | - |
| 27 | Stable | Worse | Not done | Progression | chemotherapy |

1 to 11 were the patients received osimertinib, 12 to 27 were the patients received osimertinib and bevacizumab. The patient received subsequent treatment after progression (including progression for patient with clinical response), –: continue to receive the previous treatment; Osi+ITC: osimertinib plus intrathecal chemotherapy; Osi+ WBRT: osimertinib plus whole-brain radiotherapy.

Table S2. The response for 27 patients

| Best confirmed Response | Osimertinib  n=11(%) | Osi+beva  n=16(%) |
| --- | --- | --- |
| CR | 0(0) | 0(0) |
| PR | 3(27.3) | 6(37.5) |
| SD | 6(54.5) | 7(43.8) |
| PD | 2(18.2) | 3(18.7) |

PR, partial response; SD, stable disease; PD, progressive disease.

Table S3. The extracranial metastasis of 27 patients

| Metastasis sites | Osimertinib  n=11(%) | Osi+beva  n=16(%) |
| --- | --- | --- |
| BM  Yes  No | 4(36.4)  7(63.6) | 7(43.8)  9(56.2) |
| Bone  Yes  No | 6(54.5)  5(45.5) | 12(75)  4(25) |
| Liver  Yes  No | 3(27.3)  8(72.7) | 3(25.0)  13(75.0) |
| Pleura  Yes  No  Extracranial progress  Yes  No | 4(36.4)  7(63.6)  7(63.6)  4(36.4) | 5(31.2)  11(68.8)  5(31.2)  11(68.8) |
